# Psychometric properties of the Chinese version of the aging transformation scale for older adults with dementia

**DOI:** 10.1186/s12877-020-01977-y

**Published:** 2021-01-07

**Authors:** Lai You Li, Ning Sun, Li Bo Yu, Xiao Xin Dong, Yu Chen Ying, Han Jun Lang, Li Heng Zhou

**Affiliations:** Ningbo College of Health Sciences, No 51 Xuefu Road, Yinzhou District, 315100 Ningbo City, Zhejiang Province People’s Republic of China

**Keywords:** Aging transformation scale, Surface validity, Content validity, Structural validity, Dementia

## Abstract

**Background:**

The key part of home care services is the assessment of the needs of the home environment for older adults with dementia. The present study was conducted to evaluate the psychometric properties of a newly adapted Chinese version of an instrument designed to measure aging transformation needs among older adults with dementia.

**Methods:**

A sample of 175 older adults with dementia was selected from ten communities in China. The 55-item aging transformation needs scale was answered by the participants. Content validity, Cronbach’s alpha, item-to-total correlation, and exploratory factor analysis were used to assess the reliability and validity of the instrument.

**Results:**

The aging transformation needs scale has a good surface validity, content validity, and structural validity. The content validity was 0.965; 55 items had large factor loads on their corresponding principal components (≥0.5). There was a significant correlation between the aging transformation needs scale and each component (r = 0.897–0.973, all *P*< 0.01), and 9 components also had a high correlation (r = 765~0.977, all *P*< 0.01); the total table Cronbach’s α was 0.993, the Cronbach’s α of each constituent factor was 0.944~0.990, and the correlation coefficient between factor and content was > 0.40 (all *P*< 0.01).

**Conclusions:**

Evidence was found to support the reliability and validity of the aging transformation needs scale that measures the quality of the aging transformation needs for older adults with dementia from an aging transformation needs perspective.

## Background

Dementia is a progressive degenerative disease of the central nervous system. It is the main clinical manifestation of progressive memory impairments, general mental decline, personality changes, and mental behavior abnormalities [1]. There are currently more than 35 million patients with dementia worldwide, and by 2050 this number is expected to increase to approximately 120 million. Dementia has become one of the most serious chronic diseases leading to loss of physical function [1].

Homecare involves older adults who live in their homes and not in old-age care institutions [2]. This model is currently the most important pension model in China [2]. Ten departments, including the Office of the Old Age and the Ministry of Civil Affairs, jointly issued the “Opinions on Comprehensively Promoting the Work of Home Care Services” as early as 2008. This document promoted the development of home care services [3]. The home environment is an important supporting means for home-based care services. Guaranteeing the safe and efficient development of home-based care services is necessary in order to provide a suitable living environment. An aging home environment can help older adults maximize their self-care ability and fully integrate and utilize the currently available resources. Therefore, a key part of home care services is the assessment of the needs of the home environment. At present, some home environments can no longer meet the needs of the older adults, especially those of older adults with disabilities. Many home environment designs have the following related problems: lack of elevators, unreasonable design and spatial layout, and lack of a safe bathroom space. Additionally, there is a lack of auxiliary facilities and imperfect alarm and help sign systems [4, 5]. In view of this, the author created a suitable aging transformation demand scale and evaluated its reliability and validity.

### Research status of foreign aging transformation scales

1. The Comprehensive Assessment and Solution Process for Aging Residents (CASPAR): CASPAR was developed in 2002 by a multidisciplinary team of rehabilitation, construction, management, etc. professionals in the United States. Experts designed this system to save costs and achieve retrofit assessments under extreme conditions [6]. The tool collects the daily activities and environmental related information of older adults, and assesses the needs of the home care environment, so that experts can develop appropriate transformation plans without visiting the site [6, 7]. The evaluator can also evaluate the methods through which older adults or their family members request for their care professionals such as rehabilitation teachers, medical staffs, and case managers. It also includes a small amount of on-site measurements and photographs. The entire assessment takes about 25 min [6].
  CASPAR [8] consists of 6 parts. The aging transformation scale proposes to list the care services that will be provided to older adults to match those provided by professional care services. Aging reform experts typically use the information collected via the scale to provide a transformation plan after a comprehensive analysis [6]. Several studies have shown that CASPAR has good inter-rater reliability and validity, and the correct recognition rate of environmental problems is above 88% [7]. At present, the tool is widely used in aging transformation of older adults in the United States [8] and has also been adapted for research of aged care services [9–11]. Weeks et al. [12] believe that CASPAR adopts a cooperative approach to assess the needs of the home environment transformation of older adults, which is simple and easy, but requires more time to obtain photographs and field measurements. If the scale of the living environment needs to be reduced, the photographing and field measurement can be omitted.
2. Housing Enabler: Housing Enabler was prepared by the Swedish rehabilitation engineer Iwarsson [13] in 1996 to assess the accessibility of a living environment. The tool provides a system for collecting and organizing data to provide supporting information for the aging transformation of the home environment. The tool was updated twice in 2000 and 2010, and there are currently two versions of the complete tool and the screening tool [14], and development of an internet platform based on the tool is underway. The full version of the tool has been evaluated by specially trained professionals, including personal, environmental, and score calculations [13]. Fourteen items are present in the personal part, while 12 items are used to assess whether an individual function is limited, 2 items are used to evaluate the use of mobile aids [15], the environment part consists of 3 dimensions including the external environment, entrance, and indoor environment, which are comprised of 28 entries, 46 entries, and 87 entries, respectively, for a total of 161 entries [16]. The higher the score, the greater the problem of the living environment and the stronger the demand for transformation [14]. The full version of the tool was used to conduct an inter-rater reliability test in older adults of five European countries. The results showed that the individual part-to-evaluator consistency ratio was 91%, the environmental part-evaluator agreement ratio was 85%, and the personal part kappa coefficient was 0.43. The environmental part kappa coefficient was 0.50, which proves that the reliability of the evaluators was good [17, 18]. Iwarsson et al. [19] confirmed that non-professionals were trained to use the tool screening version, and the results were highly consistent with those obtained by professionals. Because the tool is more comprehensive and systematic, it has been used in a joint study by five European countries (Germany, UK, Sweden, Hungary, Latvia). The study lasted for 3 years and investigated the relationship between the home environment and the healthy life of older adults [20].
3. The Safety Assessment of Function and the Environment for Rehabilitation Tool (SAFER): SAFER was designed in 1993 by a Canadian occupational therapist Oliver [21] for occupational therapists to implement in homes for older adults in the community. Before an environmental safety intervention, an function assessment of older adults and the home environment is necessary. Each entry in the tool combines the function of older adults with the environment and does not simply evaluate one of the two. The occupational therapist uses the tool during a home visit to score the environment based on observation, interview, and by asking older adults to complete an activity, and the entire evaluation process takes about 1 h [21]. The tool has been revised several times and the final version includes 97 entries in 14 dimensions [22]. The score is based on questions requiring “yes” or “no” answers, and the higher the score, the less suitable the current environment is for older adults [21]. The operational therapist Letts [22] tested the reliability and validity of this scoring system in 1995 and 1998. After expert evaluation, the tool has shown content validity; with a KR-20 coefficient of 0.82, a good internal consistency; both the degree and the test-retest reliability have been shown to be good [23]. In 2006, Canadian occupational therapists transformed SAFER into a more sensitive environmental intervention measurement tool, for functional and rehabilitation environmental safety assessment and for health outcome measurement and evaluation. The scope of this tool is to allow the occupational therapist to measure the suitability for older adults before and after intervention and to evaluate the effectiveness of environmental safety interventions [24]. The improved version consists of 93 entries in 10 dimensions, using a 4-level scale. The higher the score, the more serious the problem [24]. The tool has good reliability and validity, and the internal consistency is 0.859; the difference in validity is good, and it is negatively correlated with body function status, indicating that a SAFER-HOME not only measures the physical functional status, but also evaluates body function and the environment to a matching degree [24].
4. Home Assessment Profile (HAP): Designed by the American physiotherapist Chandler [25] in 2001 to assess the environmental factors that may affect the ability of older adults. The tool assesses the match between the environment and older adults by trained professionals to observe the activities of older adults in various areas of the home [25]. HAP consists of 58 items in 7 dimensions. Studies have shown that [25], after balancing other age-related factors such as age, cognitive function, and mobility, the tool can effectively predict the occurrence of falls in older adults. The higher the HAP score, the greater the likelihood of a fall. The inter-evaluator reliability was 0.92, and the test-retest reliability was 0.92 after 2 weeks [25].

### Research status of domestic aging transformation scales

With the changes in China’s social economy, population structure, and other factors, the aging process is accelerating. With such a background, Chinese scholars have included several adaptations to the living environment of older adults, the aging of the houses, and the aging of the settlements. Theoretical research and practice of aging construction and transformation design have achieved relatively fruitful results. Li Xiaoyun conducted research on urban older adults community-friendly planning strategies for older adults, and proposed an older adults friendly community social service planning strategy including housing, health and safety, old age culture, old age service system, age integration, and other planning contents such as, the living unit, friendly open spaces, friendly traffic environment, older adults service facilities, the old community suitable for aging design, and other planning content of older adults friendly community material environment planning strategy [26]. Zhou Yanqi and others analyzed the causes and performances of the current residential areas in terms of suitable aging design from different subjects such as developers, designers, and buyers. In combination with the needs of outdoor activities, the design principles for an outdoor environment suitable for older adults were proposed. The design points for different types of activity spaces, outdoor facilities and garden elements in the outdoor environment have also been addressed [27]. Yang Shenmao and others proceeded from the design concept of residential fitness and discussed the “dwelling residential design” in the United States, the “House Design Guide for Longevity Society” in Japan, and the “Residential Design for the older adults” in China. The difference between the design criteria of the three design guidelines has been previously addressed [28]. Domestic scholars have carried out most of the theoretical research and have been involved in the practice of aging construction and renovation design. There is currently no assessment of the correlation between aging and the home environment, therefore this kind of assessment is necessary in China.

In summary, because of the great differences in China’s home environment, living habits and lifestyle, and because China’s environmental and pension service professional capabilities are extremely scarce, the feasibility of directly introducing and localizing foreign assessment scales is extremely low. It is necessary to understand the status of the aging environment in China, investigate the living habits of the demented older adults in China and understand the requirements of the home environment, and compile a suitable aging assessment scale for older adults. Today, with the continuous development of community and home care services, medical staff are no longer merely continuing the basic health service functions of the community. With the deepening of the aging process, the functions of community medical staff are increasingly expanding towards compound professional skills. Medical staff should learn and use the Home Environment Assessment Scale to help design and implement a safer home environment, which will help to improve the quality of community care services.

### Purpose of the study

The purpose of this study was to evaluate the psychometric properties of the aging transformation needs scale. Using this scale, we will be able to assess the perceptions of the aging transformation needs for older adults with dementia in China.

## Methods

### Instrumentation

The aging transformation needs scale was independently created by a research team. The scale comprises 55 statements each with a five-point rating scale. Five alternative answers are given: 1 = “none,” 2 = “a little,” 3 = “some,” 4 = “many,” and 5 = “a lot.” The aging transformation needs scale consists of the following nine factors: entrance (12 items), indoor activities (6 items), toilet (3 items), bath (5 items), decoration (3 items), rest (5 items), preparation/meal (6 items), laundry (4 items), and supporting environment (11 items). The aging transformation needs scale has demonstrated good validity and reliability, and the Cronbach’s alpha coefficients ranged from 0.944 to 0.984.

### Sample

Two hundred sampling frames were provided by the older adults with dementia identified from 10 communities in five districts of Ningbo in China between August 2018 and February 2019. The sample from each district comprised 40 older adults with dementia selected using convenience sampling. A total of 200 questionnaires were distributed to older adults with dementia, and 175 questionnaires with valid responses were finally included. The inclusion criteria were as follows: a. individuals older than 60 years of age from local households; b. those who met the diagnostic criteria for dementia as per the American Diagnostic and Statistical Manual of Mental Disorders, 4th Edition, and were diagnosed by relevant specialists; and c. those who were mainly at home for care. Exclusion criteria were severe cognitive dysfunction, unconsciousness, and difficulties in communicating and understanding.

The questionnaire was returned by 188 participants (response rate: 94.0%). Thirteen questionnaires were left blank and were, therefore, excluded. The final number of analyzed questionnaires was 175.

### Ethical considerations

The Ningbo College of Health Sciences Ethics Committee approved this study (NBWY-031). Before the research was performed, all the candidates were invited to a scheduled meeting arranged by the community service center managers and were briefly informed about their right to withdraw from the study at any time. Next, a questionnaire was distributed to each patient with dementia who consented to or was authorized to participate in this study. To ensure confidentiality, each copy of the completed questionnaire was placed in an envelope with a code number for anonymity.

### Statistical analyses

In this study, the statistical analyses of the data were performed using the Statistical Package for Social Sciences software (version 22.0, SPSS Inc., Chicago, IL, USA). A *p*-value < 0.05 was considered statistically significant. Missing values were processed following the mean imputation method developed by Polit and Beck (2004). Five experts were selected to assess the aging transformation needs scale. The five experts were selected based on the following criteria: (a) having 5 years or more experience of caring for older adults with dementia; (b) working in nursing homes or community service centers; and (c) having a title of deputy senior or above. Each of the five experts selected in this study were subordinate to five community service centers located in five cities in China. Before implementation, the method proposed by Lawshe (1975) was used by an expert panel to assess the content validity of the questionnaire. A content validity ratio (CVR) was introduced in this study for quantitative assessment. In the formula, $\mathrm{CVR}=\frac{ne-N/2}{N/2}$, ne and N represent the number of panelists who indicated “fairly relevant” or “very relevant” in response to a specific question in the questionnaire and the total number of panelists, respectively. The relevance of the whole questionnaire was assessed by each of the five selected experts. One of the following four possible answers were selected: do not need it at all, do not need it, need it somewhat, or need it totally. The exploratory factor analysis and principal component analysis were performed with varimax rotation as the method of extraction to determine the construct validity of the questionnaire and derive an independent sub-questionnaire out of the total 55-item questionnaire, respectively. Four factors were chosen to evaluate the constructed sub-questionnaire when a loading level of 0.50 was used as a threshold for including items. The internal consistency of the instrument was tested by calculating the Cronbach’s alpha coefficients of the entire questionnaire and each of the sub-questionnaires (Polit & Beck, 2004). The potential correlation between items was assessed by performing an item-total correlation analysis at an accepted threshold of correlation coefficient = 0.40 (Spector, 1992).

## Results

All of the subjects suffered from dementia and their ages ranged from 60 to 98 years old, with an average age of 78.67 years. The monthly income ranged from 1000 to 8000 yuan, with an average income of 3546.68 yuan. There were 98 females (56.0%) and 77 males (44.0%). Most patients had a primary school education degree (*n* = 108, 61.7%). Most subjects were widowed (*n* = 100, 57.1%).

According to the meaning of the aging transformation needs scale and its constituent factors, five experts analyzed the degree of consistency between the nine constituent factors and their descriptions. The results are shown in Table 1.

**Table 1:** Evaluation of the surface validity of the aging transformation needs scale (items)

Factors	Very relevant	Related but still needs to be changed	Must be modified to be relevant	Irrelevant	Total	The number of items
Import and export demand	55	3	2	0	60	12
Indoor activity needs	28	2	0	0	30	6
Toilet needs	13	1	1	0	15	3
Bathing needs	24	1	0	0	25	5
Modifying needs	13	1	1	0	15	3
Rest needs	22	3	0	0	25	5
Meal needs	28	2	0	0	30	6
Laundry needs	19	1	0	0	20	4
Supporting Environmental needs	50	3	2	0	55	11
Total	252	17	6	0	275	55

The CVR between the expert panelists ranged from 0.8 to 1.0. The results of the last question concerning the relevance of the total aging transformation needs scale showed that the five administrators unanimously agreed that the total scale was relevant.

To determine the validity of the aging transformation needs scale, a factor analysis was conducted which was deemed appropriate given the Kaiser–Meyer–Olkin (KMO) measure of 0.93 and the significant Bartlett test (X^2^ = 5739.316; *P*< 0.0001). A KMO value of 0.93 indicated that the sample size was sufficient for the factor analyses and the significant results of the Barlett test showed that the correlation matrix of the scale items was appropriate for the factor analyses. To determine the factor structure of the ageing transformation needs scale, a PCA with a varimax rotation was conducted. Nine factors, accounted for 95.2% of the variance. The factor load test adopts the maximum variance rotation method, and the entries of the 55 scales have large factor loads on their corresponding principal components (all ≥0.5). The factor analysis results suggest that the home environment suitable for the aging transformation needs scale has a higher structural validity.

The aging transformation needs scale scores are shown in Table 2. Overall, the patients highly perceived their aging transformation needs [mean = 26.84; standard deviation (SD) = 4.59]. The subjects believe that they had the greatest access to rest needs (mean = 4.48, SD = 0.81) and access to supporting environmental needs (mean = 4.47, SD = 0.78).

**Table 2:** Detailed description of the aging transformation needs scale (*n* = 175)

The scale and its sub- scale	Mean	Standard deviation	Score range	The number of items
Import and export demand	4.29	0.71	1–5	12
Indoor activity needs	4.44	0.76	1–5	6
Toilet needs	4.41	0.79	1–5	3
Bathing needs	4.42	0.80	1–5	5
Modifying needs	4.41	0.88	1–5	3
Rest needs	4.48	0.81	1–5	5
Meal needs	4.40	0.86	1–5	6
Laundry needs	4.35	0.92	1–5	4
Supporting environmental needs	4.47	0.78	1–5	11
Total scale	26.84	4.59	9–45	55

The aging transformation needs scale and its various factor correlation scales presented a high correlation with the nine factors (both *P* < 0.01). The specific results are shown in Table 3.

**Table 3:** Correlation between the aging transformation needs scale and its various factors (r)

Factors	Import and export demand	Indoor activity needs	Toilet needs	Bathing needs	Modifying needs	Rest needs	Meal needs	Laundry needs	Supporting environmental needs
Total scale	0.897^**^	0.921^**^	0.906^**^	0.962^**^	0.941^**^	0.953^**^	0.948^**^	0.936^**^	0.973^**^
Import and export demand		0.905^**^	0.765^**^	0.834^**^	0.794^**^	0.790^**^	0.766^**^	0.765^**^	0.800^**^
Indoor activity needs			0.784^**^	0.867^**^	0.856^**^	0.863^**^	0.780^**^	0.831^**^	0.838^**^
Toilet needs				0.859^**^	0.808^**^	0.851^**^	0.918^**^	0.835^**^	0.901^**^
Bathing needs					0.952^**^	0.911^**^	0.911^**^	0.894^**^	0.932^**^
Modifying needs						0.928^**^	0.860^**^	0.911^**^	0.914^**^
Rest needs							0.905^**^	0.900^**^	0.951^**^
Meal needs								0.912^**^	0.977^**^
Laundry needs									0.922^**^
Supporting environmental needs									

The internal consistency coefficient (Cronbach’s alpha) was 0.99 for the total questionnaire and varied between 0.95 and 0.99 for the nine sub-scales. In the item analysis, the correlations ranged from 0.40 to 0.99. The items of the supporting sub-scale had the highest correlations. All of the 55 items had a corrected item-total correlation > 0.40 (Table 4).

**Table 4:** Reliability and correlations between the items and the total aging transformation needs scale (*n* = 175)

Items	Cronbach’ s α	Item-to-total correlation
Import and export demand	0.94	0.40~0.90
Indoor activity needs	0.95	0.84~0.95
Toilet needs	0.99	0.99~0.98
Bathing needs	0.98	0.89~0.99
Modifying needs	0.98	0.98~0.99
Rest needs	0.96	0.84~0.97
Meal needs	0.99	0.96~0.99
Laundry needs	0.98	0.97~0.98
Supporting environmental needs	0.98	0.76~0.97
Total scale	0.99	0.47~0.97

## Discussion

### Current status of the aging transformation needs scale

Table 1 shows that the number of entries that were “very relevant” and “relevant, but needed to be changed” (269/275), and the “one-point irrelevant” entry was 0. This result indicated that the home environment was suitable. Therefore, the aging transformation demand scale has a good surface validity. The CVR of the Home Environment Adaptation Requirement Scale was 0.965, which meets the requirements of CVR ≥ 0.80. These results proved that the self-made items of this scale basically met the requirements for suitable aging transformation needs; however, there was also a “must be modified to be relevant” item with a proportion of 2.18% (6/275). This result prompted us to further analyze the data and pay attention to the individual differences of the patients themselves.

Table 2 shows that the care needs of the respondents were focused on resting and the presence of a supporting environment and indoor activity space. In most cases, people with mild dementia could eat, wear, and wash, among others. These patients were able to take care of themselves in their daily life and reduce their dependence on others. Therefore, there is a high demand for transforming the supporting environment and indoor activity space, with the hope to create larger and safer spaces for activities. Older adults often request for lights near the bed as the indoor lighting allows them to see their surroundings. Additionally, phone and doorbell sounds should be loud and the flooring should be anti-skid to prevent falls among older adults. This is consistent with the research results of An Haoyuan [5]. A large number of studies conducted in China and abroad [29, 30] found that the home environment of older adults is important to improve their quality of life. In addition, the aging transformation of the home environment can help prevent falls, delay functional decline, and save manpower costs. Therefore, it is necessary for home care services to give importance to the needs of environmentally-friendly aging transformations. Demand assessment is the basis for this transformation, and we hope that the government and relevant personnel will pay close attention to these needs and provide the required support.

Table 3 shows that there was a significant correlation between the aging transformation needs scale and the nine constituent factors. The nine constituent factors of the scale also had a good correlation. The results of this study indicate that if relevant departments actively explore the needs of the aging population, this will greatly improve the living environment of the older adults with dementia and consequently, their survival. Quality promotes the rapid development of China’s pension unemployment.

### Reliability of the aging transformation needs scale

The questionnaire had a good reliability. Reliability is defined as the degree of reliability and stability of a scale, expressed in terms of reliability coefficients. Reliability is a prerequisite for questionnaire design [31]. The Cronbach’s alpha is an important indicator of reliability in a questionnaire, and is used to assess the internal consistency of the sub-sections in the full scale and their respective reliabilities [32]. The scale designed here was deemed reliable and valid, and the items in the scale were relevant to the aging transformation needs of older adults with dementia. In addition, an item-to-total correlation analysis was performed to validate the scale’s reliability [33]. The high item-total and test-retest correlation coefficients further showed the reliability of the scale. Repeatability was not assessed on this scale because it requires two measurements of the same object using the same scale in a short period of time. Considering the large sample size of this study and the special group analyzed, it was not appropriate to complete two measurements of the patients in a short period of time.

### Validity of the aging transformation needs scale

The scale had a good validity. Validity is defined as the extent to which a measurement tool can measure the event it is measuring. CVRs ranging from 0.8 to 1.0 were obtained from the expert panelists. All five experts agreed that the full scale was relevant. The items loaded on the nine factors were in line with each sub-scale. The high factor loadings of items on each factor were consistent with Waltz et al.’s recommendation of at least 0.4 for factor loadings [34]. It is acceptable that individual factors could explain 3.2 to 64.6% of the variance. The explanation of 95.2% of the variance by the nine factors was consistent with the recognized standard that an ideal factor analysis explains 40 to 60% of the variance. Our factor analysis results were consistent with the original nine-factor model. This evaluation further shows that the questionnaire is valid, reliable, and suitable for the use as an aging transformation needs measurement for older adults with dementia.

### Limitations and further research

The psychometric testing was limited by the use of a convenience sample and the fact that the data gathering sites were located in ten communities in one city in China. In the future, a test–retest procedure could be used to further establish the reliability of the long-term care needs questionnaire. As a result of the large sample, it was not feasible to examine the test–retest reliability. Therefore, further research is required to verify the relationships between the aging transformation needs and aging transformation needs outcomes such as life quality and cognitive status in the same way researchers have studied these relationships in Western countries. Further studies with older adults with dementia in China are also needed to evaluate the utility of the aging transformation needs scale.

## Conclusion

Research on the aging of the home environment has just started in China, and the aging reform is very important to improve the quality of life of older adults. However, there is a lack of understanding of older adults themselves and most of older adults professionals. The development of a home environment suitable aging transformation demand scale is imperative. The key feature of this research is the comprehensive use of multiple methods to construct the conceptual framework of the scale. The use of content analysis to analyze the existing survey research results to obtain the conceptual framework of the scale, and then the data obtained through interviews method to compare the scale. This method is an innovation of the method of constructing the scale framework.

The development of the self-evaluation home environment suitable aging transformation demand scale can help older adults self-assess the degree of matching between the living environment and their own functional status, thus finding reasonable and scientific transformation needs. At the same time, community and home care services can also use this scale to more economically and conveniently assess the needs of the living environment, reduce the risk of falls, delay dysfunction, and improve the quality of life. With the continuous development of community and home care services and the gradual deepening of the aging process, higher requirements are placed on the development of composite professional skills of community medical staff. Medical staffs should learn and use the assessment scale for the aging transformation of the home environment in order to assist builders in designing and implementing a safe home environment and contribute to the improvement of the quality of community care services.

## Data Availability

The data sets generated and analyzed during the current study are not publicly available due to ethical restrictions and patient confidentiality but are available from the corresponding author on reasonable request. The aggregated data are provided in the tables.

## Abbreviations

DSM-IV: American Diagnostic and Statistical Manual of Mental Disorders, 4th Edition
CVR: Content validity ratio
PCA: Principal component analysis
SD: Standard deviation
KMO: Kaiser–Meyer–Olkin
